# Non-Coding RNA Analyses of Seasonal Cambium Activity in *Populus tomentosa*

**DOI:** 10.3390/cells11040640

**Published:** 2022-02-11

**Authors:** Huimin Xu, Bo Chen, Yuanyuan Zhao, Yayu Guo, Guijun Liu, Ruili Li, Viktoria V. Zeisler-Diehl, Yanmei Chen, Xinqiang He, Lukas Schreiber, Jinxing Lin

**Affiliations:** 1 College of Biological Sciences, China Agricultural University, Beijing 100193, China; xuhm@cau.edu.cn (H.X.); chenyanmei@cau.edu.cn (Y.C.); 2 College of Biological Sciences and Biotechnology, Beijing Forestry University, Beijing 100083, China; chenbo_1991@bjfu.edu.cn (B.C.); yyzhao@bjfu.edu.cn (Y.Z.); guoyau1510@bjfu.edu.cn (Y.G.); liruili@bjfu.edu.cn (R.L.); 3 Institute of Tree Development and Genome Editing, Beijing Forestry University, Beijing 100083, China; 4 College of Life Sciences, Peking University, Beijing 100871, China; hexq@pku.edu.cn; 5 Institute of Radiation Technology, Beijing Academy of Science and Technology, Beijing 100875, China; lliuguijun@brc.ac.cn; 6 Department of Ecophysiology, Institute of Cellular and Molecular Botany, University of Bonn, Kirschallee 1, 53115 Bonn, Germany; v.zeisler@uni-bonn.de (V.V.Z.-D.); lukas.schreiber@uni-bonn.de (L.S.)

**Keywords:** lncRNA, circRNA, cambium activity periodicity, *Populus tomentosa*

## Abstract

Non-coding RNA, known as long non-coding RNA (lncRNA), circular RNA (circRNA) and microRNA (miRNA), are taking part in the multiple developmental processes in plants. However, the roles of which played during the cambium activity periodicity of woody plants remain poorly understood. Here, lncRNA/circRNA-miRNA-mRNA regulatory networks of the cambium activity periodicity in Populus tomentosa was constructed, combined with morphologic observation and transcriptome profiling. Light microscopy and Periodic Acid Schiff (PAS) staining revealed that cell walls were much thicker and number of cell layers was increased during the active-dormant stage, accompanied by abundant change of polysaccharides. The novel lncRNAs and circRNAs were investigated, and we found that 2037 lncRNAs and 299 circRNAs were differentially expression during the vascular cambium period, respectively. Moreover, 1046 genes were identified as a target gene of 2037 novel lncRNAs, and 89 of which were the miRNA precursors or targets. By aligning miRNA precursors to the 7655 lncRNAs, 21 lncRNAs were identified as precursors tof 19 known miRNAs. Furthermore, the target mRNA of lncRNA/circRNA-miRNA network mainly participated in phytohormone, cell wall alteration and chlorophyll metabolism were analyzed by GO enrichment and KEGG pathway. Especially, circRNA33 and circRNA190 taking part in the phytohormone signal pathway were down-regulated during the active-dormant transition. Xyloglucan endotransglucosylase/hydrolase protein 24-like and UDP-glycosyltransferase 85A1 involved in the cell wall modification were the targets of lncRNA MSTRG.11198.1 and MSTRG.1050.1. Notably, circRNA103 and MSTRG.10851.1 regulate the cambium periodicity may interact with the miR482. These results give a new light into activity–dormancy regulation, associated with transcriptional dynamics and non-coding RNA networks of potential targets identification.

## 1. Introduction

Meristems with the constant ability for new organs or tissues are playing important roles in plant development [1,2]. As a lateral meristem, composed of fusiform initials and ray initials, the vascular cambium decides the secondary growth in plant, dividing to produce secondary phloem to the outside and secondary xylem to the inside. In boreal forest, the cambium of trees exhibits a seasonally cyclical pattern of activity and dormancy, providing fuel wood and carbon sequestration. Over the past decade, the molecular basis of vascular cambium activity related with activity-dormancy cycles (vascular cambium periodicity) have been investigated [3,4], the regulation of non-coding RNA networks on vascular cambium periodicity was still unknown. 

Non-coding RNA was specific expression and producing functional RNA molecules rather than encoding proteins to involve in different stages or tissues, which can be classified as small RNAs (sRNAs) (<200 nt), long non-coding RNAs and circular RNA [5]. MicroRNAs with length of 20 to 24 nt are known to play important spatiotemporally posttranscriptional regulatory roles in plants [6,7]. CircRNAs could be preventing the miRNAs to bind with their target genes, which act as cytoplasmic miRNA sponges [8,9,10,11] and regulators of nuclear transcription [12]. 

It has been reported that non-coding RNAs were involved in the cellular differentiation, epigenetic regulation, gene regulation and other various development stages [13,14,15,16]. In some herbaceous plants, many lncRNAs were identified and reported, such as the licRNA responsive to biotic and/or abiotic stresses in *Arabidopsis* [14] and long noncoding natural antisense transcripts (IncNATs) dynamically correlated with the histone acetylation by light-responsive expression changes [15]. There were also various investigations of lncRNA in rice [17,18] and maize [19,20,21]. In woody plants, putative lncRNAs of potential functions in wood formation in *Populus tomentosa*, including cellulose and lignin biosynthesis [22], and endogenous hormone regulation of the tension wood formation in *Catalpa bungei* [23]. Another investigation revealed that lncRNAs of both lncWOX5 and lncWOX11, which are involved in the formation and development in poplar adventitious roots were found [24]. It has been reported that the drought-responsive of lncRNAs and analysis the regulatory function in *Populus trichocarpa* [10], and GA-responsive lncRNAs with the functional analysis of targets by participating in auxin signal transduction and synthesis of cellulose and pectin in *Populus tomentosa* [25]. Moreover, differentially expressed lncRNAs of *Populus euphratica* with heteromorphic leaves in response to environment stress [26], stage-and tissue-specific expression patterns of lncRNAs in *Ginkgo biloba* [27,28], and some novel ncRNAs in Chinese fir were identified [29]. Furthermore, cell/tissue specific and developmental specific expression of circRNAs were also investigated [9,30,31]. In particular, considerable researches on the cambial activity throughout the activity-dormancy cycle based on and transcriptome miRNA expression changes [32,33,34,35], and also age-dependent miRNAs regulation [36]. Although numerous works of non-coding RNAs in trees were reported, the regulation mechanisms of the cambial meristem during the dormancy-active periodicity, especially the lncRNA multiple effects combined with circRNAs and miRNAs were unclear.

In this research, we first comprehensively investigated the regulation of lncRNAs and circRNAs in vascular cambium periodicity of *P. tomentosa* from nine RNA-seq libraries at a genome-wide scale, and focused on the lncRNAs and circRNAs expressed at active, transition and dormant stages, as well as some related miRNAs. This research would enable us to increase systems-level of the molecular regulation mechanisms of vascular cambium development.

## 2. Materials and Methods

### 2.1. Plant Materials

Vascular cambium was obtained from the stem of poplar trees, *Populus tomentosa*. The trees, almost 3-year-old, are growing naturally in Guanxian County, Shandong Province, China. Cambium materials were separated from wood blocks which were obtained on 3 January, 20 March, and 22 June 2015. The wood blocks were put into liquid nitrogen immediately and the isolation of vascular cambium from wood blocks was referred to the method described in our previous research [33,37]. 

### 2.2. Cytological Observations and Periodic Acid Schiff (PAS) Staining

Different vascular cambium samples with active, transition and dormant stages of 4 mm^3^ from stem of poplar trees (1.3 m above the ground) were collected and fixed with 2.5% glutaraldehyde in 100 mM phosphate buffer (pH 7.2). After serial dehydration with ethanol and acetone, the entrapment of different samples with Spurr’s resin were conducted following serial dehydration by using ethanol and acetone. Toluidine blue staining was performed with 0.25% weight to volume ratio (*w*/*v*) toluidine blue O (Sigma, MO, USA) after semi-thin sectioning (2 μm), and polysaccharide staining was performed with Periodic Acid Schiff Reagent (PAS) for 30 min before microscopic observation. 

### 2.3. LncRNA and CircRNA Library Construction and RNA-Sequencing 

According to the manufacturer’s instructions of TRK-1001 reagent (LC Sciences, TX USA), the total RNA of vascular cambium was obtained from *P. tomentosa*. RNA integrity number (RIN) greater than 7.0, and of which 260/280 nm ratio between 1.8 to 2.0 were used for the analysis. Total 10 µg RNA was used to deplete ribosomal RNA (rRNA) according to the manuscript of the Epicentre Ribo-Zero Gold Kit (Illumina, SD, USA). Following purification, the poly(A)- or poly(A)+ RNA fractions is fragmented into small pieces by divalent cations with elevated the temperature. Then the cleaved RNA fragments were reverse-transcribed to create the final cDNA library for the Illumina HiSeq 4000 Sequencing at LCBIO, HangZhou, China. Also, the sequence assembly of RNA-seq data to the reference genome of *P. trichocarpa* v3.0, https://phytozome.jgi.doe.gov/pz/portal.html (accessed on 17 January 2015).

### 2.4. Expression Profiling and Identification of mRNA, lncRNA and circRNA

Firstly, the transcripts shorter than 200 bp were discarded, which overlapped with known mRNAs and transcripts. Transcripts with coding potential were furtherly to predicted by utilizing CPC, CNCI and Pfam [38,39,40], removing all transcripts with CPC score < −1 and CNCI score < 0.

Using the FPKM using StringTie software v1.0.0, http://ccb.jhu.edu/software/stringtie/ (accessed on 17 January 2015), mRNAs, lncRNAs, and circRNAs expression levels were calculated by The mRNAs, lncRNAs, and circRNAs was determined differentially expression by the standard of “|log_2_ (fold change)| ≥ 1” and “*p* value < 0.05” [41]. 

### 2.5. Prediction and Functional Analysis of Target Genes of lncRNAs and circRNAs

LncRNAs were reported to regulate neighboring target genes by playing a cis role. The target genes of the lncRNA was considered as the protein-coding genes located 100 kb upstream and downstream of a lncRNA [23].The cis-target genes of lncRNAs from different samples were predicted in order to further explore the function of lncRNAs.In current research, GO and KEEG pathway analysis of the target genes for lncRNAs during the vascular cambium development by using the Blast2GO v3.1, https://www.biobam.com/download-blast2go/?cn-reloaded=1 (accessed on 18 August 2015) at *p* value < 0.05 levels [42]. Moreover, the lncRNA/circRNA-miRNA-mRNA network was constructed using Cytoscape software v3.7.1, https://github.com/cytoscape/cytoscape/releases/3.7.1/ (accessed on 4 January 2019).

### 2.6. LncRNA Identified as miRNA Precursors and Predicted to Be miRNA Targets

To explore the lncRNAs were aligned with precursors of known miRNAs, we used the miRBase v21.0, https://www.mirbase.org/ftp/21/ (accessed on 29 October 2015) using BLAST with default parameters. The lncRNA homologous to miRNA precursors with > 90% coverage was eventually defined as miRNA precursors [40,43]. The program ‘miRPare’ was used to predict novel miRNA precursors from the lncRNAs. The lncRNAs and miRNAs were submitted to ‘psRNATarget’, http://plantgrn.noble.org/psRNATarget/ (accessed on 29 October 2017) with an expectation ≤ 3. Base the no more than four mismatches and G/U pairs within the lncRNAs and miRNAs complementary regions were considered to be miRNA targets [40,43]. Lacting as precursors of known or novel miRNA during the activity-dormant transition of *P. tomentosa* were analyzed using the method reported in previous study [10,40]

## 3. Results

### 3.1. Cytological Observation and Periodic Acid Schiff (PAS) Staining 

**Figure 1:**
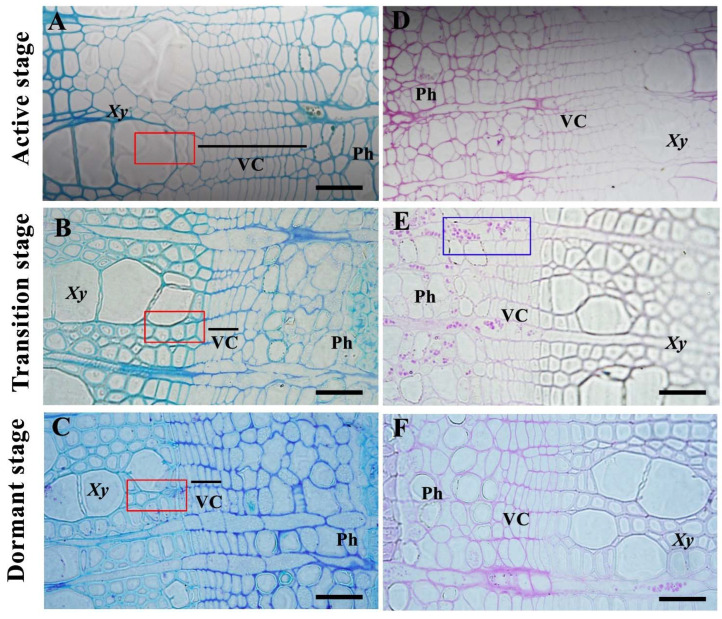
Morphology observation and polysaccharide analysis in the cambium of *P. tomentosa*. (**A**–**C**) active, transition, and dormant cambium stages. (**D**–**F**) distribution of polysaccharides in cambium and phloem at the three stages. Scale bars: 100 μm. The red boxes show the developing xylem outside of the cambium and the blue box show the polysaccharides. Xy, Xylem; Ph, Phloem; VC, Vascular cambium.

In current research, the cambium was divided into active, transition and dormant stages, based on morphology analysis. During the active-dormant stage, the number of cell layers was increased and the cell wall was much thicker (Figure 1A–C). We also found that the cell wall of developing xylem outside of cambium was much thicker during the transition from active to dormant stage. In order to evaluate the cell division and physiological state during cambial zone, we examined the polysaccharide changes firstly by using Periodic Acid Schiff (PAS) staining, and found that the polysaccharide was the most abundant in transition stages (Figure 1D–F), indicating that the initiation of cambium and phloem maybe gathers the energy for dormancy. Taken together, these results showed evident anatomical features of the active-dormant cycles existed during vascular cambium periodicity.

### 3.2. Overview Analysis of mRNA and lncRNA in Vascular Cambium

To determine transcriptional and post-transcriptional changes that occur during vascular cambium development, we used vascular cambium from stem of active stage (VC1), transition stage (VC2) and dormant stage (VC3) for high-throughput RNA-seq. nine strand-specific libraries from three biological replicates of different stages (VC1, VC2 and VC3) were constructed by RNA-Seq. High-quality clean reads were obtained by removing the adaptor and unqualified data, and finally, we found that over 97, 277, 364–151, 086, 708 raw sequence reads and 14.59–22.66 G raw bases for each replicate in Table 1. More than 92, 964, 890–146, 426, 386 clean reads and 13.94–21.96 G clean bases were obtained throughout raw data trimming. The percentage of the GC content are 45.5–46.5%, 49–51% and 53%, respectively. The Q20 proportion value for all replicates was more than 99.72% (Table 1). 

**Figure 2:**
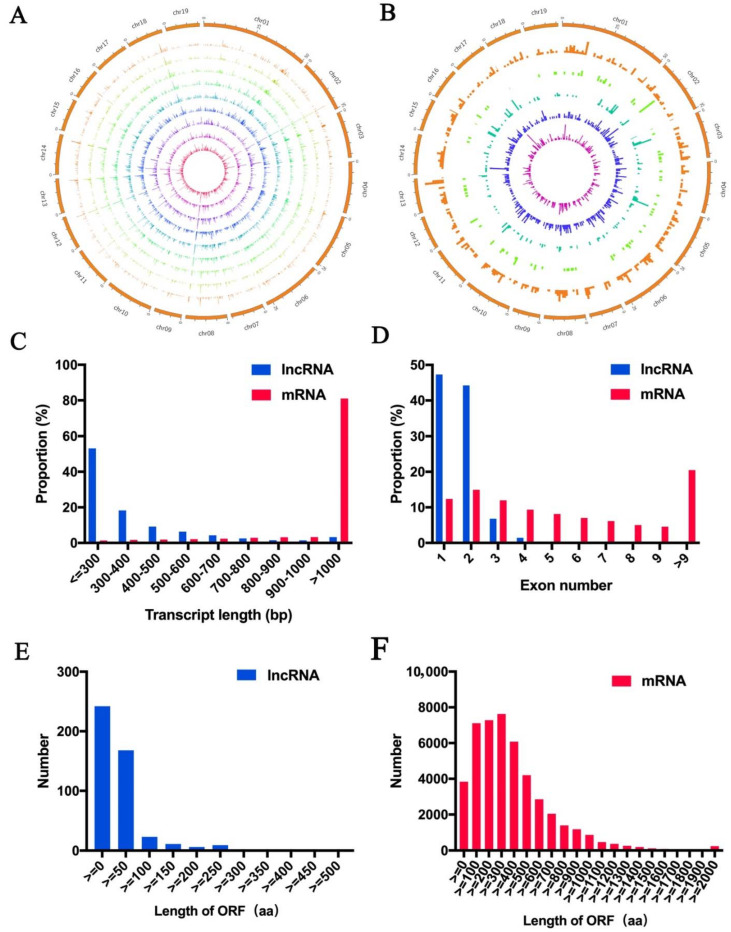
Expression patterns of lncRNAs and mRNAs. (**A**) lncRNA density distribution with nine samples expression, representing VC1-1, VC1-2, VC1-3, VC2-1, VC2-2, VC2-3, and VC3-1, VC3-2, VC3-3 from center to the outside. (**B**) lncRNA density distribution with five class code expression, representing u, o, x, j and i from center to the outside. (**C**) protein-coding transcripts length distribution and lncRNAs. (**D**) protein-coding transcripts exon number and lncRNAs. (**E**) ORF length in lncRNAs. (**F**) ORF length of protein-coding transcripts.

LncRNA density distribution with sample expression and class code expression were showed in Figure 2A and Figure 2B, of which 82% of protein-coding transcripts sequence length were longer than 1000 bp, 2.1% lncRNAs was longer than 1000 bp (Figure 2C). lncRNAs contained fewer exons than the protein-coding transcripts. Moreover, open reading frames (ORFs) length in lncRNAs were less than 100 aa, which was shorter than that in protein-coding transcripts (Figure 2E–F), indicating the differential expression pattern between protein-coding transcripts and lncRNAs.

**Figure 3:**
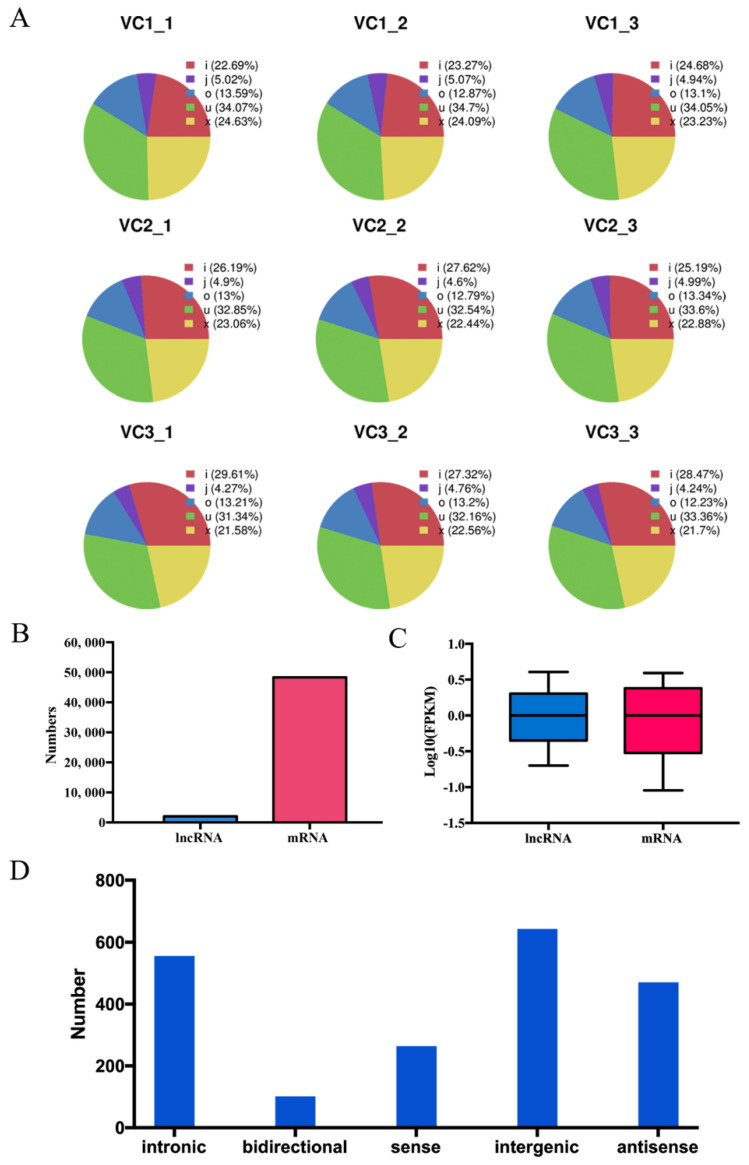
Characteristics of the non-coding RNAs. (**A**) Distribution of six types of lncRNAs. Known lncRNAs as for class code “=, with orange”), intronic lncRNAs as for class code “i, with light green”, lncRNAs share with reference at least one splice junction as for class code, with dark green, lncRNA of generic exonic overlap with a reference transcript as for class code “o, with blue”, lncRNA as for class code “u, with violet” and antisense lncRNA as for class code “x, with pink”. (**B**) Expression numbers of the mRNA-lncRNA expression. (**C**) Expression levels lncRNAs and mRNAs. (**D**) The numbers of five types of lncRNA.

Furthermore, all potential lncRNAs in current research were assembled by Stringtie. Coding Potential Calculator (CPC) and Coding-Non-Coding-Index (CNCI) were used to eliminate potential coding transcripts. Accordingly, there were five categories of lncRNAs identified and named as ‘i’, ‘j’, ‘o’, ‘u’, and ‘x’, which represents intronic, bidirectional, sense, intergenic, and antisense lncRNA, respectively. (Figure 3A). The mRNA-lncRNA expression level and number were also showed in Figure 3B and Figure 3C. To further obtain putative lncRNAs, the class code annotated by Cufflinks v2.2.1, http://cole-trapnell-lab.github.io/cufflinks/releases/v2.2.1/ (accessed on 5 May 2014) were used to filter the transcripts. Finally, 2037 reliably expressed lncRNAs (FPKM >1 for one library) were obtained, including 556 intronic lncRNAs, 102 bidirectional lncRNA, 264 sense lncRNAs, 644 intergenic lncRNAs, and 471 antisense lncRNAs (Figure 3D). 

### 3.3. CircRNA in Vascular Cambium 

CircRNAs locations antisense to known transcripts was arising from intronic, intergenic, untranslated regions and ncRNA loci, which are primarily derived from the exons of protein-coding genes [11,43]. In our investigation, at active stage, 70.51–85.1% of identified circRNAs were from exons of a protein-coding gene, 0.71–1.15% and 11.22–28.34% were from introns and intergenic regions, respectively. Additionally, in transition stage, 76.11–86.28%, 1.21–1.54% and 12.18–222.45% were from exons of a protein-coding gene, introns, and intergenic regions. Moreover, in dormant stage, 60.93–64.84%, 1.65–1.76% and 33.40–33.51% were from exons of a protein-coding gene, introns, and intergenic regions (Figure S1). These results indicated that the identified circRNAs were predominantly exonic circRNAs, and derived from different genomic regions and mainly from coding regions, especially in active stage. 

### 3.4. Different Expression Analysis of mRNAs, lncRNAs and circRNAs

Here, the expression levels of genes, lncRNAs and circRNAs during the cambium activity periodicity were investigated, by using the fragments per kilobase per million mapped reads (FPKM) value. Amounts of protein-coding transcripts were higher expressed than lncRNAs (Figure 3). In addition, genes, lncRNAs or circRNAs with|log_2_ (fold change)| ≥ 1 at *p*-value < 0.05 were considered as differentially expressed. 

**Figure 4:**
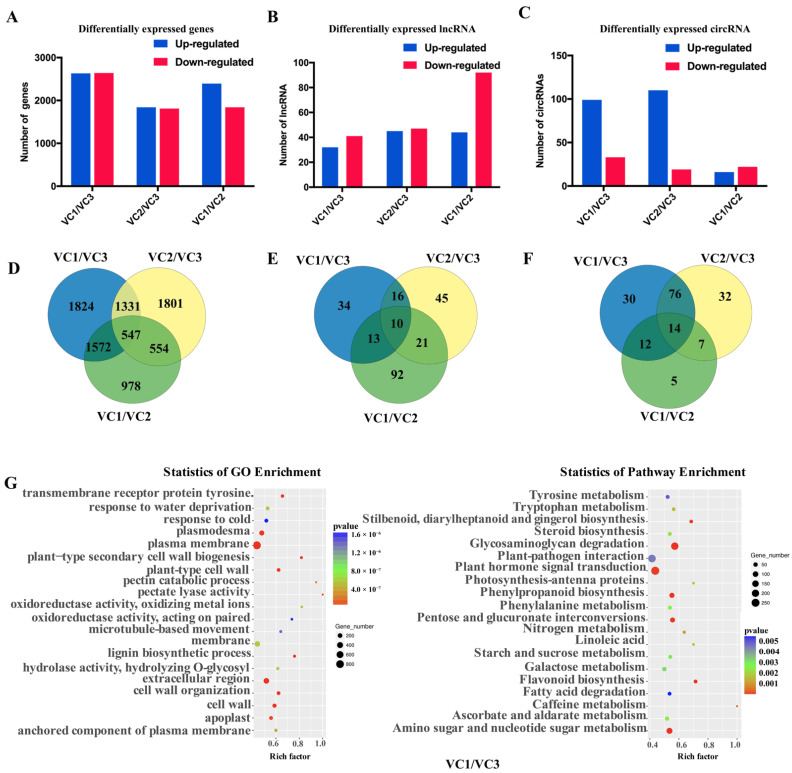
The analysis of differentially expressed genes, lncRNAs, and circRNAs. (**A**) numbers of differentially expressed genes. (**B**,**C**) numbers of differentially expressed lncRNAs and circRNAs. (**D**–**F**) differentially expressed genes, lncRNAs and circRNAs by Venn diagram. (**G**) GO and KEGG pathway enrichment of differentially expressed genes.

In our results, there were 2633 upregulated and 2641 downregulated of 5274 DEGs from VC1 vs. VC3,1840 upregulated and 1811 downregulated of 3651 DEGs from VC2 vs. VC3, 2393 upregulated and 1840 downregulated of 4233 DEGs from VC1 vs VC2 (Figure 4A,C, Table S1). The numbers of DEGs specific and common to each comparison group were illustrated using a Venn diagram (Figure 4). In addition, 32 upregulated and 41 downregulated lncRNAs were identified in the comparison of VC1 vs. VC3. Moreover, 45 upregulated and 47 downregulated lncRNAs were found in VC2 vs. VC3, while 44 upregulated and 92 downregulated were identified in VC1 vs. VC2 (Figure 4B,E). Additionally, the all cis-regulated lncRNAs were 36 with 19 differentially cis-regulated in VC1 vs. VC2. The levels of the 299 differentially expressed circRNAs during the vascular cambium period were compared. By comparison of VC1 and VC3, upregulated and downregulated circRNAs were 99 and 33, respectively. 110 circRNAs and 19 circRNAs were found upregulated and downregulated specifically in VC2 vs. VC3. Moreover, 16 upregulated and 22 downregulated circRNAs were found in VC1 vs. VC2 (Figure 4C,F).

**Figure 5:**
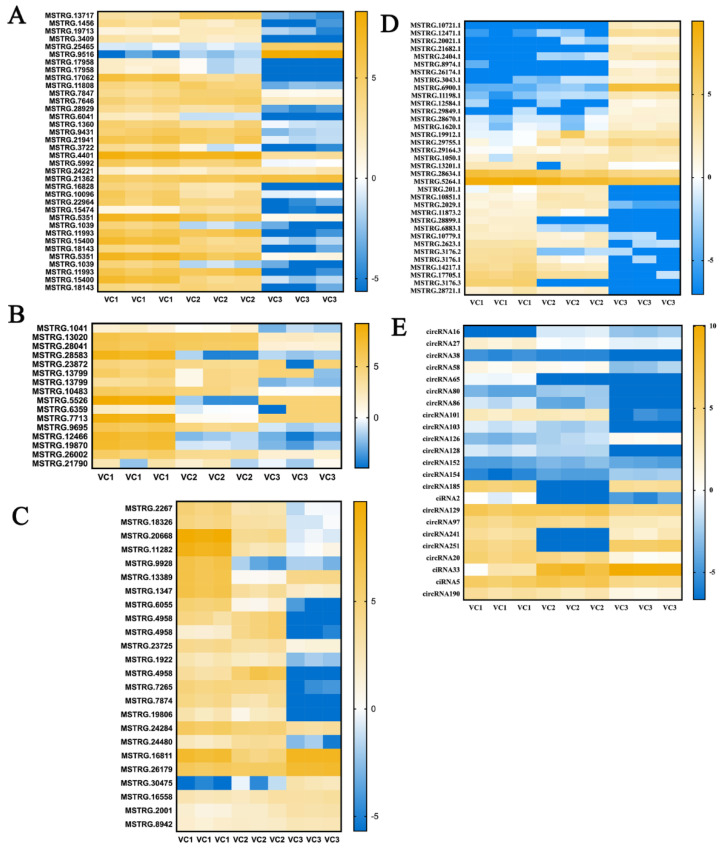
The analysis of differentially expressed genes and non-coding RNAs. (**A**) The differentially expressed genes of phytohormone signal transduction, (**B**) starch and sucrose metabolism, and (**C**) phenylpropanoid biosynthesis. (**D**) The differentially expressed candidate lncRNAs that interacted with the miRNAs and mRNAs. (**E**) The differentially expressed candidate circRNAs that interacted with the mRNAs and miRNAs. Different colors denote different expression levels, with white representing low expression and blue representing high expression. The values beside the colors represent log_2_(FPKM + 1).

Overall, the total number differentially expressed lncRNAs harbored was less than DEG. The differentially expression level of lncRNAs during cambium development were shown in the heatmap (Figure 5, Table S2). We found that the expression of genes encoding these proteins of auxin response factor 7-like, auxin-induced protein 22A, auxin-induced protein IAA4 and auxin-responsive family protein were all up-regulated comparing the active and dormant stages (Figure 5A, Table S2). MSTRG.13717 (DELLA protein GAI-like) showed the higher expression in the active stage (Figure 5A, Table S2). In addition, the lncRNA MSTRG.953 (zinc finger family protein) related with cell division was up-regulated in VC1 vs VC3 (15.81 fold). In plant hormone signal transduction pathway, the hypothetical protein (e.g., MSTRG.21903) and serine/threonine-protein kinase CTR1-like (MSTRG.12789) were down-regulated in VC1 vs. VC2 (−1.36 fold and −1.55fold), and VC1 vs. V3 (−1.3 fold and −1.45 fold), respectively. 

Additionally, Photosynthesis-antenna proteins (MSTRG.1600), Porphyrin and chlorophyll metabolism (MSTRG.26475), MSTRG.18307 (DnaJ heat shock family protein) was down-regulated in VC1 vs VC2 (−1.26 fold). We further found that the cellulose synthases (e.g., MSTRG.1041, MSTRG.13020, MSTRG.28041, MSTRG.28583) and xyloglucan endotransglucosylase/hydrolase protein (MSTRG.5351, MSTRG.1039, MSTRG.11993, MSTRG.15400) were all up-regulated with higher levels in active stages (Figure 5B). Moreover, mannitol dehydrogenase 1 family protein (MSTRG.2267), cinnamoyl-CoA reductase 1-like (MSTRG.18326), Chain A family protein (MSTRG.20668) and cytochrome P450 98A3 family protein (MSTRG.11282) were also up-regulated in active stage. Some hypothetical protein (MSTRG.1922, MSTRG.4958, MSTRG.7265, MSTRG.7874, MSTRG.19806, MSTRG.24284) mainly taking part in phenylpropanoid biosynthesis were also evident differentially expressed (Figure 5C), indicating that cell wall undergoes dynamic modification during the seasonal changes. There were many other novel lncRNAs with unknown function and several circRNA (Figure 5D,E), such as circRNA33, circRNA190 involved in plant hormone and circRNA241 related with cell wall were evident up-regulated in active stage comparing the transition stages with the dormant stage, which may be imperative for the cambium activity. 

### 3.5. Potential Functional Roles for lncRNAs and circRNAs

Gene expression usually regulated by LncRNAs, which acting on neighboring target genes, known as the cis-acting role of lncRNAs. Here, putative cis-target genes of lncRNAs by searching for coding genes were transcribed within 100 kb upstream or downstream. In total, 2037 novel lncRNAs with 1046 predicted target genes were identified in vascular cambium of *P. tomentosa.*, and a number of the differentially expressed target genes were identified mainly enriched in plasma membrane (GO:0005886), carbohydrate metabolic process (GO:0005886), vacuole (GO:0005773), cell wall (GO:0005618), defense response (GO:0006952) and “protein phosphorylation” (GO:0006468) by GO enrichment analysis (Figure 4 and Figure S2). Furthermore, the KEGG analysis showed that a number of target genes of starch and sucrose metabolism (ko00500), phenylpropanoid biosynthesis (ko00940), Fructose and mannose metabolism (ko00051), pentose and glucuronate interconversions (ko00040), glycosaminoglycan degradation (ko00531), glycerolipid metabolism (ko00561), plant-pathogen interaction (ko04626) and plant hormone signal transduction (ko04075), taking part in the seasonal environment changes, especially response to the light and temperature environment (Figure 4 and Figure S2). These findings indicated that the differentially expressed lncRNA-mRNA interaction pairs may participate in plant hormones and photosynthesis pathways which could be involved in the cambial activity.

### 3.6. LncRNA with Potential Target Genes

**Figure 6:**
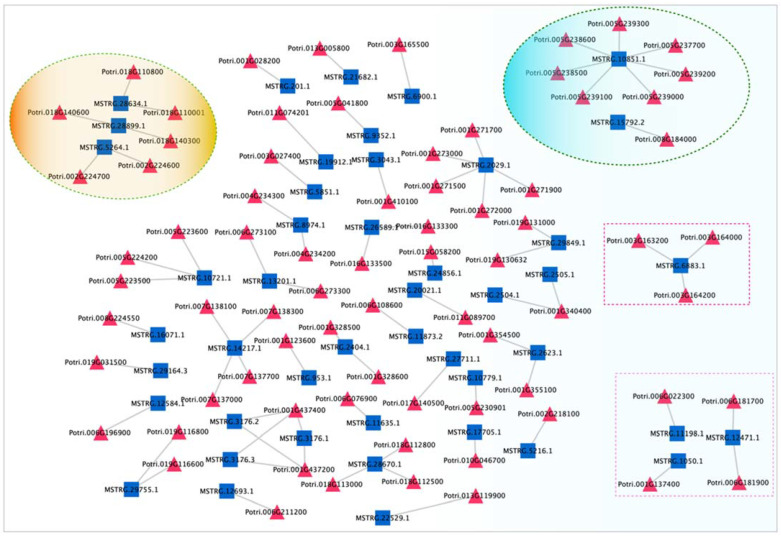
Regulatory networks of candidate lncRNAs with target mRNAs. Blue squares indicate lncRNAs, red triangles indicate the target mRNAs.

By cytoscape, here, we constructed lncRNAs and their potential target genes network (Figure 6). As shown in Figure 5, one lncRNA may have epistatic interactions with 1–7 potential target genes and the same genes can be targeted by the different lncRNA. We discovered 9 lncRNAs including MSTRG.16029.2, MSTRG.8532.3, MSTRG.8534.1, MSTRG.44394.1, MSTRG.61045.1, MSTRG.61046.1, MSTRG.36258.1, MSTRG.36259.1, and MSTRG.8532.1, targeting nine photosynthesis-related mRNAs. For example, glycosyl hydrolase family 9 protein (Potri.005G237700), hexokinase (Potri.005G238600), chlorophyll a-b binding protein 2 (Potri.005G239200, Potri.005G239300), WEB family protein (Potri.005G238500) which was the targets of MSTRG.1050.1 related with the photosynthesis. Moreover, xyloglucan endotransglucosylase/hydrolase protein 24-like (Potri.001G137400) and UDP-glycosyltransferase 85A1 (Potri.006G022300) involved in the cell wall modification were the targets of MSTRG.11198.1 and MSTRG.1050.1, respectively. Furthermore, cellulose synthase-like protein D5 (Potri.006G181700 and Potri.006G181900) were as the targets of the MSTRG.12471.1, implying that the cell wall expansion and elongation were dynamic during the active -dormant transition.

In addition, it was specific patterns in the progress of genes related plant hormones during the vascular cambium period. In spring, the reactivation of cambium needed the energy from catabolism of sucrose and fats, whereas activation of the pentose phosphate shunt could reduce equivalents. ARF GTPase-activating domain-containing family protein (Potri.003G16320), *p*-(*S*)-hydroxymandelonitrile lyase-like (Potri.003G164000), nuclear-pore anchor (Potri.003G164200) involved in the plant hormones were the targets of MSTRG.6883.1 gibberellin regulated protein (Potri.005G239000), gibberellin-regulated GAST/Snakin family protein (Potri.005G239100), which was the targets of MSTRG.1050.1, showing that gibberellin (GA) signaling and biosynthesis could be necessary in the regulation of the activity-dormant transition.

### 3.7. LncRNA/circRNA-miRNA-mRNA Network Analysis

Considering that lncRNAs to some extent was considered as precursors of known miRNAs, in current investigation, the lncRNA sequences to determine if they could be precursors or targets of known miRNAs were examined. In this investigation, there were 21 lncRNAs identified as precursors to 19 known miRNAs, by aligning miRNA precursors to the 7655 lncRNAs (Table 2). For example, the lncRNA (MSTRG.28721.1) was predicted as the precursor of ptc-MIR396b. Other 18 known miRNAs were also identified, such as ptc-MIR156g ptc-miR159b, ptc-MIR160f, ptc-MIR166c, ptc-MIR168a, ptc-MIR172c, ptc-MIR172e, ptc-MIR172h, ptc-MIR319c, ptc-MIR319d, ptc-MIR394b, ptc-MIR395i, ptc-MIR396d, ptc-MIR475b, ptc-MIR475d, ptc-MIR482a, ptc-MIR1450, ptc-MIR6438b in *P. tomentosa* (Table S2). Also, there were seven genes as the targets for MSTRG.10851.1, including Potri.005G237700.1, Potri.005G238600.1, Potri.005G239000.2, Potri.005G239100.2, Potri.005G238500.1, Potri.005G239200.1, Potri.005G239300.1 (Figure 6).

**Figure 7:**
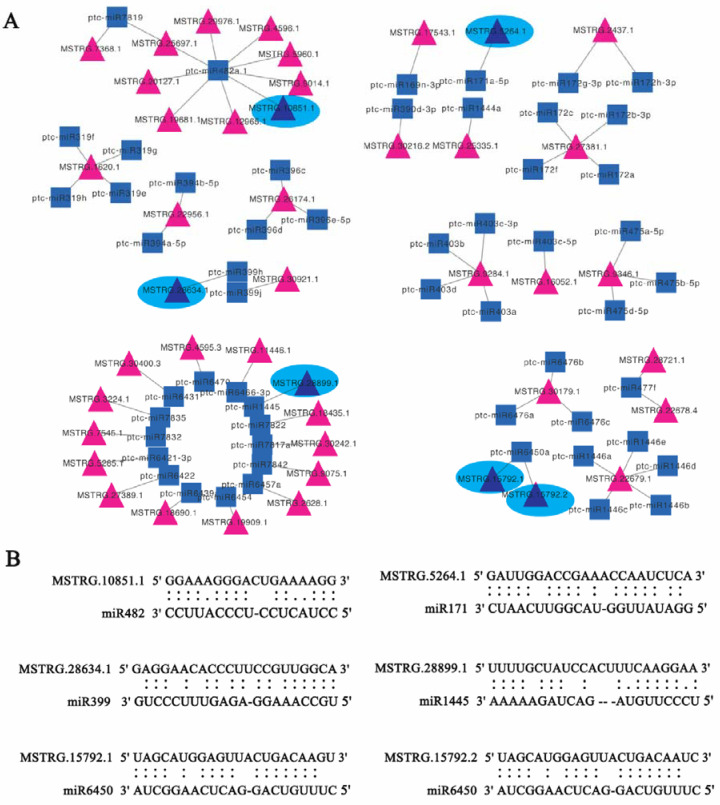
Non-coding RNA transcripts and the interaction analysis. (**A**) lncRNAs and miRNAs with interaction networks. (**B**) The lncRNAs targeted by miRNAs. Blue squares indicate miRNAs, red triangles indicate the lncRNA transcripts.

Importantly, the sequences of some miRNAs and lncRNAs could be no more than four mismatches and G/U pairs within the lncRNAs and miRNAs complementary regions, further comfirming that miRNAs may target lncRNAs. Our results furtherly showed that 41 lncRNAs related with 49 target genes were also identified to be targeted by miRNAs. As shown in Figure 7A, there were 9 lncRNAs as targets by the miR482, such as MSTRG.10851.1, MSTRG.12965.1, MSTRG.19681.1, MSTRG.20127.1, MSTRG.25697.1, MSTRG.29976.1, MSTRG.4596.1, MSTRG.5960.1, MSTRG.9014.1. In addition, PagHAP2-6 was regulated by ABA involved in cambium dormancy in our previous research [34], here, MSTRG.17543.1 as the target of ptc-miR169n-3p was targeting the PagHAP2-6. MSTRG28634.1 with the targets Potri.018G110800 and Potri.018G110001, which was also targeted by ptc-miR399h. Furthermore, some lncRNAs were targeted by miRNA1445(MSTRG28899.1), miRNA171(MSTRG5264.1) and miRNA6450(MSTRG16792.1, MSTRG16792.2) associated with their own targets (Figure 4 and Figure 7). Importantly, MSTRG.1620.1 as the targets for miR319 family (ptc-miR319e, ptc-miR319f, ptc-miR319g, ptc-miR319h) and MSTRG.26174.1 as the targets of miR396 family (ptc-miR396c, ptc-miR396d, ptc-miR396e-5p). Moreover, some other lncRNAs as the targets for miRNAs also have various targets genes. For example, miR399 (MSTRG28634.1), miRNA1445(MSTRG28899.1), miRNA171(MSTRG5264.1) and miRNA6450 (MSTRG16792.1, MSTRG16792.2) (Figure 7B). Notably, our findings also revealed that circRNA103 as target of miR482, which increased 7.12-fold and 6.05-fold during the VC1 vs VC3 and VC1 vs VC3, respectively. The findings revealed the interaction of lncRNA-miRNA-circRNA networks are involved in in the dynamic cambium activity during the active-dormant cycles.

**Figure 8:**
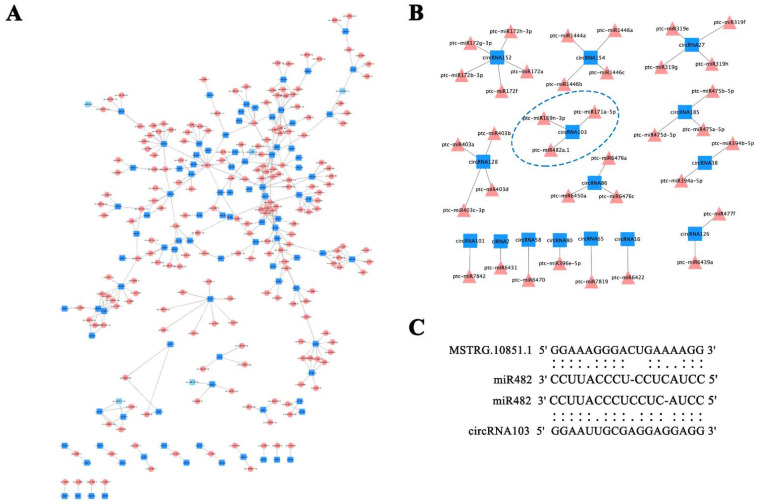
The circRNAs as miRNA targets. (**A**) lncRNAs and miRNAs with interaction networks. (**B**) CircRNAs targeted by miRNAs. (**C**) The interaction of circR103, miR482, and MSTRG.10851.1.

**Figure 9:**
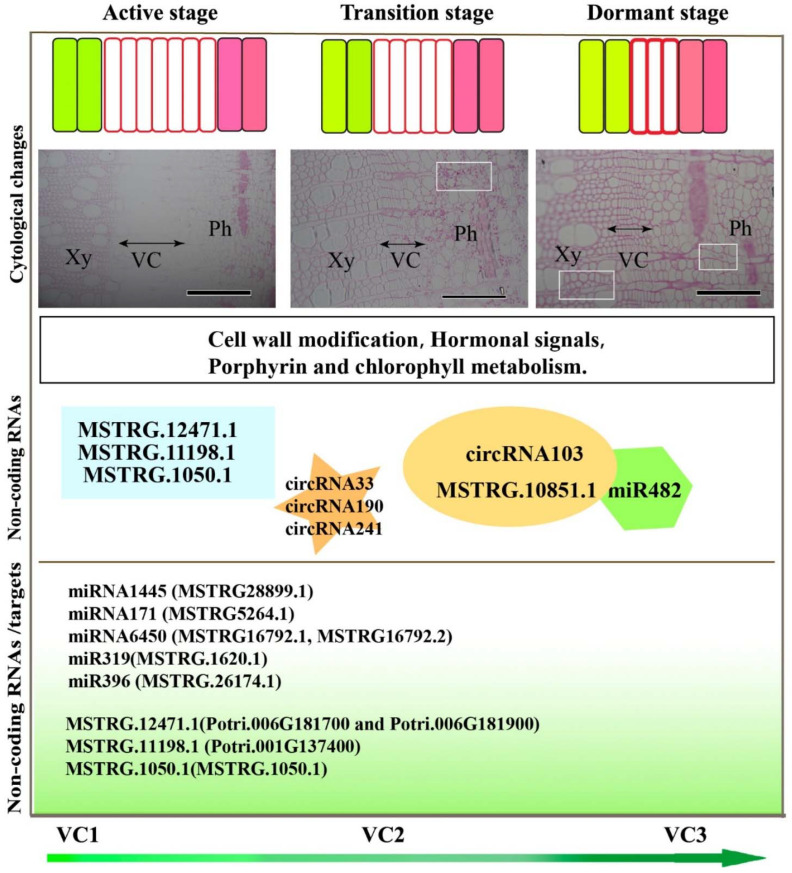
Diagram of the universal regulatory mechanisms of non-coding RNAs in secondary meristems in *P. tomentosa* during the active-dormant transition. The cambium cell numbers and cell wall thickness are modified, which is related to gene expression and cytology. Non-coding RNA regulation of gene expression contributes to cell wall modification, hormone signals, and porphyrin and chlorophyll metabolism, for example, lncRNAs MSTRG.12471.1, MSTRG.11198.1, and MSTRG.1050.1; and circRNAs circRNA33, circRNA190, and circRNA241. circRNA103 interacted with MSTRG.1050.1 and miR482. The non-coding RNAs and their targets are shown in the diagram. The light microscopy images are from Figure 1. Xy, Xylem; Ph, Phloem; VC, Vascular cambium. Bars in the microscopy images represent 200 μm.

Specifically, MSTRG.10851.1 was also identified as the target of miR482 during transition from dormancy to active growth (Figure 8). Based on previously published findings and these results, we propose a conceptual model for regulatory mechanisms of non-coding miRNAs in secondary meristem in *P. tomentosa* during the active-dormant transition (Figure 9). These results demonstrated that there was intimate connection and the complexity of the interactions among the miRNA-mRNA-lncRNA, which may provide important roles during the active-dormant transition in woody plants.

## 4. Discussion

Researches on meristem activity were constantly increasing, there is growing evidence indicating that lncRNA, miRNAs, and circRNAs are taking part in multiple biological processes, such as expression levels, and conservative properties with the high throughput sequencing technology [13,44,45,46]. LncRNAs-miRNA-circRNA playing key roles in secondary meristem tissues were identified to understand their role for meristem seasonal activity and the adaptive mechanism of woody plants. Intergenic lncRNAs act as siRNAs precursor molecules and expressed under the stress conditions were also investigated [19,24]. It has also been reported that lncRNA and miRNA as sponges or decoys to improve the secondary wall and wood biosynthesis genes [47]. In black tea, some lncRNAs interacted with 698 mRNAs were found as endogenous target mimics of miRNAs, and several lncRNAs are predicted to be taking part in the aroma formation [31]. Our results showed that 2037 novel lncRNAs with 1046 predicted target genes may regulate the vascular cambium of *P. tomentosa* during the active-dormant transition. Moreover, lncRNAs length were shorter than transcripts and ORFs, fewer exons and lower expressed compared with mRNAs were reported in several previous researches [24,26,46].

In the present research, our analysis revealed that many protein-coding transcripts were targeted by lncRNAs play key roles in multiple biological progress, such as cell wall modification, photosynthesis and metabolic pathway. We all known that cellulose was the important composition of the plant cell wall, and small-interfering RNAs (siRNAs) derived from 3′-coding region of the cellulose synthase gene superfamily, regulating the cell wall synthesis by natural cis-antisense pairs in *Hordeum vulgare* [48]. In *Arabidopsis*, CESA1, CESA3, and CESA6 interact to form CSCs that involved in primary wall biosynthesis [49,50,51]. Inhibition of cellulose synthase-like protein D5 expression changes in cell wall composition of *Cunninghamia lanceolate* were investigated in our previous research [52]. Our current results showed that cellulose synthase A family (Potri.001G136200, Potri.006G251900, Potri.018G029400, Potri.018G103900) and cellulose synthase-like (Csl) families (Potri.007G076500, Potri.007G076500, Potri.005G194200, Potri.002G257900, Potri.003G097100, Potri.004G059600, Potri.005G087500, Potri.006G181900, Potri.011G069600, Potri.016G054900) were all up-regulated in active stage compared to the dormant stage, implying that the cell wall was undergoing various reorganization during the vascular cambium period. We further found that Potri.006G181900(cellulose synthase-like protein D5) with higher expression in active stage as the targets of the lncRNA MSTRG.12471.1. Additionally, it was reported that plant cell wall could be loosened by xyloglucan endotransglucosylase activity [53], which conformed by our current results that xyloglucan endotransglucosylase/hydrolase protein 24-like and UDP-glycosyltransferase 85A1 with higher expression in active stage. Particularly, these two genes were identified as the targets of MSTRG.11198.1 and MSTRG.1050.1. Thus, the lncRNA MSTRG.12471.1, MSTRG.11198.1 and MSTRG.1050.1. Identified in this research were discerned to affect the cell wall alteration in vascular cambium.

LncRNA was not encoding proteins, revealing the potential function of lncRNAs by the gene expression specificity [23,29,31]. LncRNAs can also be as miRNA precursors, or be targeted by miRNAs may function. It has been reported that eight lncRNAs were targets of miRNAs in Maize and IPS1 regulating its expression by miR399 in *Arabidopsis* [54]. In another study, two rice lncRNAs as targets of miR160 and miR164 were reported [55]. In current research, we identified 89 novel lncRNAs, of which may as harbor miRNA precursors or be targets of miRNAs. We further constructed a comprehensive network of RNA-mediated interactions, including lncRNA-mRNA, miRNA-lncRNA, miRNA-circRNA, the lncRNA/ circRNA-microRNA-mRNA network interactions. It has been indicated that the key roles of miRNAs in regulating plant development and biological process. Earlier studies on miR403 on plant defenses and play an important role to integrate complex environmental stimuli [56]. miR164, miR396 and miR319 were reported to be function in cell proliferation [57,58,59,60]. Our previous study revealed that miR167 and miR390 involved in the auxin pathway, or GA signal pathway in active growth by expression level of miR159 [34]. In current research, we found that MSTRG.1620.1 as the targets for miR319 family (ptc-miR319e, ptc-miR319f, ptc-miR319g, ptc-miR319h). Combined with our previous result that the TCP family transcription factor (TCP) which was the targets of miR319 related with wood formation [61], here, MSTRG.1620. 1 was speculated to be involved in the different cambium stages. Altogether, these results revealed that the lncRNA/ circRNA-microRNA-mRNA network interacted function in the active-dormant cycles.

The circRNAs act as cytoplasmic miRNA sponges and sequester miRNAs has been reported previously [8,31,46], as well as a result of cis-transcriptional promotion of circRNAs on their parent genes [12]. Here, our results revealed that ~80.83% circRNAs were mainly derived from exons, promoting to produce the proteins by circRNA translation. The UDP-glycosyltransferase 85A1 (Potri.006G022300) involved in the cell wall modification was the target of MSTRG.10851.1. We further analysed the circRNA103-MSTRG.10851.1-miR482 interact network. Overall, our current research suggested the potential role of non-coding RNA in regulating the transition from active to dormant. 

## 5. Conclusions

In conclusion, combined with the light microscopy and Periodic Acid Schiff (PAS) staining, we revealed that evident changes in cell walls thickness and polysaccharides. In addition, a number of non-coding RNAs mainly including lncRNA and circRNA were identified and characterized. Totally, 2037 novel lncRNAs and 299 differentially expressed circRNAs were identified in our study. We further found that 2037 novel lncRNAs with 1046 predicted target genes and 89 of which may as harbor miRNA precursors or be targets of miRNAs. GO and KEGG analysis showed that many non-coding RNAs take part in phytohormone transduction, cell wall alteration and chlorophyll metabolism. We further constructed the regulatory networks of lncRNA/circRNA-miRNA-mRNA, such as circRNA103 and MSTRG.10851.1 may interact with the miR482 regulating the cambium periodicity. Overall, our results bring new horizons into the dynamics of transcriptional and non-coding RNA networks in the regulation of the activity-dormancy in cambium. 

## Figures and Tables

**Table 1:** Statistical Data of the RNA-Seq Reads for Nine Cambium Libraries in *P. tomentosa*.

Sample	Raw Data		Clean Data		Valid Ratio (Reads)	Q20 %	Q30 %	GC Content %
	Read	Base	Read	Base				
**VC1_1**	1,124,44,458	16.87G	111,485,756	16.72G	99.15	99.74	96.30	46.50
**VC1_2**	122,463,514	18.37G	121,394,546	18.21G	99.13	99.72	96.56	47.50
**VC1_3**	112,389,280	16.86G	111,043,910	16.66G	98.80	99.77	96.71	45.50
**VC2_1**	115,127,118	17.27G	111,956,130	16.79G	97.25	99.79	95.17	51
**VC2_2**	97,277,364	14.59G	92,964,890	13.94G	95.57	99.74	94.71	51
**VC2_3**	100,517,156	15.08G	97,028,536	14.55G	96.53	99.80	95.32	49
**VC3_1**	149,169,180	22.38G	143,203,980	21.48G	96.00	99.76	94.86	53
**VC3_2**	151,086,708	22.66G	146,426,386	21.96G	96.92	99.78	95.04	53
**VC3_3**	120,006,274	18.00G	116,163,014	17.42G	96.80	99.72	94.41	53

**Table 2:** LncRNAs Identified as Precursors of Known miRNAs in *P. tomentosa*.

Query ID	Subject ID	% Identity	Alignment Length	Mismatches	Qs	Qe	*E*-Value	Score
MSTRG.27554.1	ptc-MIR156g	100	103	0	153	255	9 × 10^−55^	204
MSTRG.24747.1	ptc-MIR159b	100	187	0	33	219	2 × 10^−105^	371
MSTRG.25598.1	ptc-MIR160f	100	100	0	96	195	1 × 10^−53^	198
MSTRG.4923.1	ptc-MIR166c	100	102	0	1	102	8 × 10^−55^	202
MSTRG.5960.1	ptc-MIR168a	100	160	0	105	264	6 × 10^−89^	317
MSTRG.19118.1	ptc-MIR172c	100	128	0	355	482	1 × 10^−69^	254
MSTRG.16341.1	ptc-MIR172e	100	133	0	491	623	1 × 10^−72^	264
MSTRG.14761.1	ptc-MIR172h	100	143	0	47	189	2 × 10^−78^	283
MSTRG.22860.1	ptc-MIR319c	100	196	0	1529	1724	7 × 10^−110^	389
MSTRG.29863.1	ptc-MIR319d	100	193	0	69	261	6 × 10^−109^	383
MSTRG.10920.1	ptc-MIR394b	100	147	0	948	1094	7 × 10^−81^	291
MSTRG.26642.1	ptc-MIR395i	100	102	0	230	331	2 × 10^−54^	202
MSTRG.28721.1	ptc-MIR396b	100	132	0	190	321	4 × 10^−72^	262
MSTRG.28721.2	ptc-MIR396b	100	132	0	190	321	7 × 10^−72^	262
MSTRG.11547.1	ptc-MIR396d	100	166	0	653	818	2 × 10^−92^	329
MSTRG.15924.4	ptc-MIR475b	100	136	0	862	997	4 × 10^−74^	270
MSTRG.15292.1	ptc-MIR475d	100	103	0	988	1090	2 × 10^−54^	204
MSTRG.15298.1	ptc-MIR482a	99.07	107	1	100	206	6 × 10^−55^	204
MSTRG.8827.1	ptc-MIR1450	99.34	151	1	554	704	6 × 10^−81^	291
MSTRG.9195.1	ptc-MIR6438b	96.86	223	7	296	518	7 × 10^−110^	387
MSTRG.9195.2	ptc-MIR6438b	96.86	223	7	197	419	6 × 10^−110^	387

## Data Availability

Not applicable.

## Supplementary Materials

The following supporting information can be downloaded at: https://www.mdpi.com/article/10.3390/cells11040640/s1, Table S1: Different Genes LncRNAs, CircRNAs, 2022-2-5; Table S2: Heatmap of Genes and Noncoding RNAs-2022-2-5. Figure S1: Sequence features of circRNAs at different stages. Figure S2: GO and KEGG pathway enrichment of differentially expressed target genes of differentially expressed lncRNAs. Figure S3: The lncRNA-microRNA-mRNA network at the cambium. Green nodes represent miRNAs, blue nodes represent lncRNAs and pink nodes represent mRNAs. Figure S4: The circRNA-microRNA-mRNA network during the cambium.
