# Supplementary material for: Non-Coding RNA Analyses of Seasonal Cambium Activity in Populus tomentosa
Source: Cells. 2022 Feb 11;11(4):640. doi: 10.3390/cells11040640 (PMC8869787; doi:10.3390/cells11040640)
Supplement: Supplementary file 1 [file cells-11-00640-s001.zip › cells-1554938-Figures.pdf]

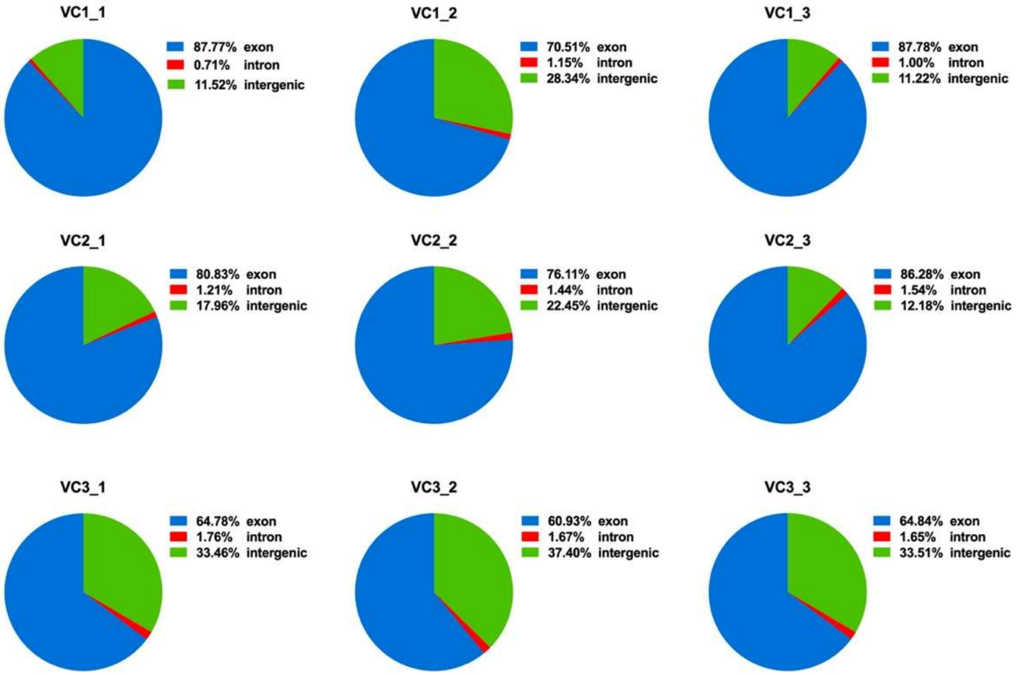

Figure S1 Sequence features of circRNAs at different stages. (a) exonic circRNAs that contained different number of exons derived from parental genes.

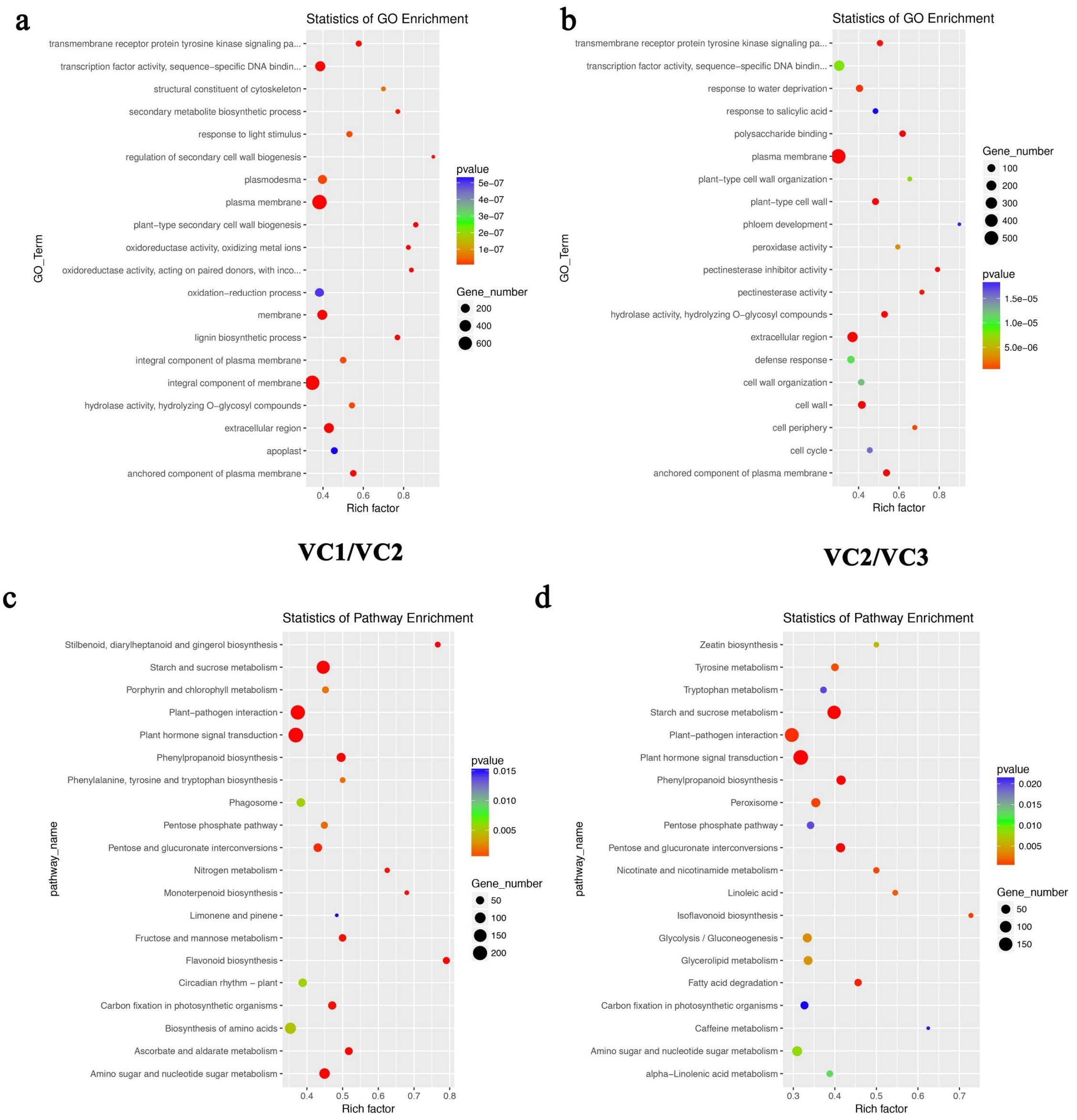

Figure S2. GO and KEGG pathway enrichment of differentially expressed target genes of differentially expressed lncRNAs. (a)(b) the KEGG pathway enrichment of differentially expressed genes during the VC1/VC2 and VC2/VC3, respectively. (c)(d)the KEGG pathway enrichment of target genes of differentially expressed lncRNAs during the VC1/VC2 and VC2/VC3, respectively.

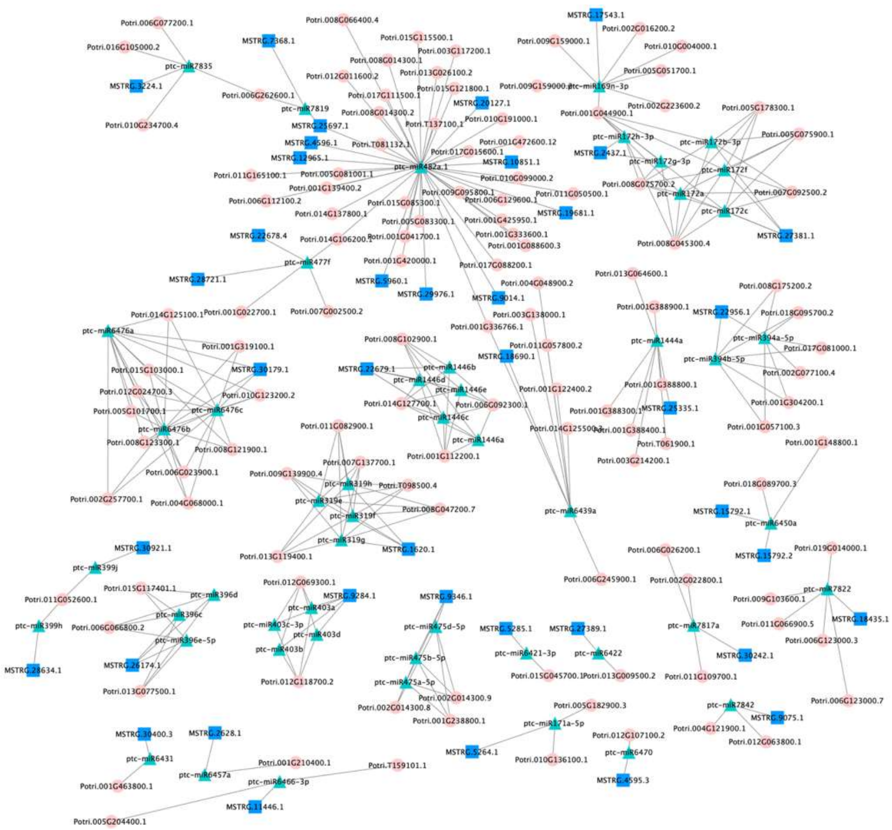

Figure S3 The lncRNA-microRNA-mRNA network at the cambium. Green nodes represent miRNAs, blue nodes represent lncRNAs and pink nodes represent mRNAs

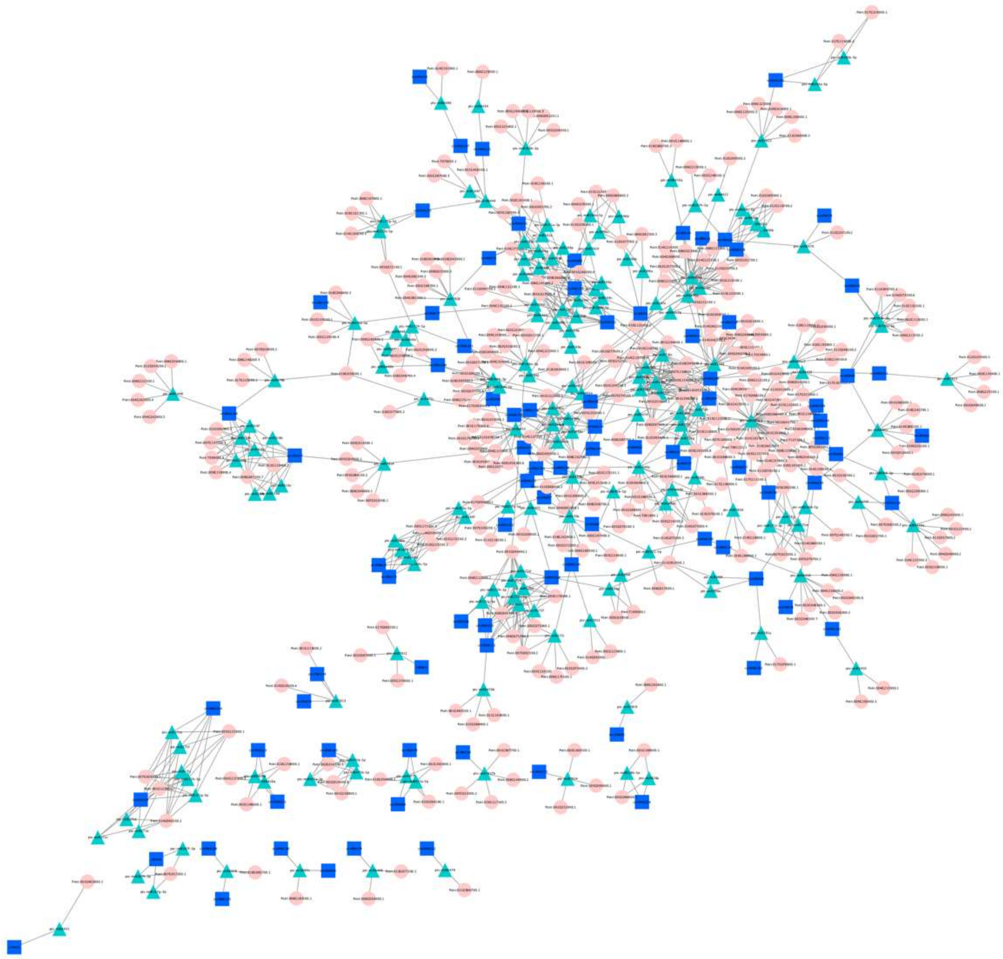

Figure S4 The circRNA-microRNA-mRNA network during the cambium.
